# Novel Therapeutics and the Path Toward Effective Immunotherapy in Malignant Peripheral Nerve Sheath Tumors

**DOI:** 10.3390/cancers17142410

**Published:** 2025-07-21

**Authors:** Joshua J. Lingo, Elizabeth C. Elias, Dawn E. Quelle

**Affiliations:** 1 Cancer Biology Graduate Program, University of Iowa, Iowa City, IA 52242, USA; joshua-lingo@uiowa.edu; 2 Department of Neuroscience and Pharmacology, University of Iowa, Iowa City, IA 52242, USA; 3 UI-MARC Program, University of Iowa, Iowa City, IA 52242, USA; 4 Department of Microbiology and Immunology, Carver College of Medicine, University of Iowa, Iowa City, IA 52242, USA; 5 Department of Pathology, Carver College of Medicine, University of Iowa, Iowa City, IA 52242, USA; 6 Holden Comprehensive Cancer Center, University of Iowa, Iowa City, IA 52242, USA

**Keywords:** MPNST, targeted therapy, immunotherapy

## Abstract

Malignant Peripheral Nerve Sheath Tumors (MPNSTs) are a deadly subtype of soft tissue sarcoma for which effective therapeutic options are lacking. Currently, the best treatment for MPNSTs is complete surgical resection with wide negative margins, but this is often complicated by the tumor size and location and/or the presence of metastases. Radiation or chemotherapy may be combined with surgery, but patient responses are poor. Targeted treatments, including small-molecule inhibitors of oncogenic proteins such as mitogen-activated protein kinase kinase (MEK), cyclin-dependent kinases 4 and 6 (CDK4/6), and Src-homology 2 domain-containing phosphatase 2 (SHP2), are promising therapeutics for MPNSTs, especially when combined together, but they have yet to gain approval. Immunotherapeutic approaches have been revolutionary for the treatment of some other cancers, but their utility as single agents in sarcoma is limited and not approved for MPNSTs. The immunosuppressive niche of MPNSTs is thought to confer inherent treatment resistance, particularly to immunotherapies. Remodeling an inherently “cold” tumor microenvironment into a “hot” immune milieu to bolster the anti-tumor activity of immunotherapies is of great interest throughout the cancer community. This review focuses on novel therapeutics that target dysregulated factors and pathways in MPNSTs, as well as different types of immunotherapies currently under investigation for this disease. We also consider how certain therapeutics may be combined to remodel the MPNST immune microenvironment and thereby generate a durable anti-tumor immune response to immunotherapy.

## Simple Summary

Sarcomas are rare but deadly cancers of the connective tissues and bone. Malignant Peripheral Nerve Sheath Tumors (MPNSTs) are sarcomas that arise from Schwann cells in the trunks and extremities. They are aggressive tumors that lack effective therapies. Immunotherapies can provide durable anti-cancer activity for some cancers, but they have shown limited efficacy in sarcomas, including MPNSTs. Currently, there is great interest in identifying targeted therapies, such as small-molecule kinase inhibitors, that halt MPNST proliferation and survival. Interest in such drugs rises if their actions lead to the remodeling of the tumor immune landscape to bolster the response to immunotherapy. Here, we explore key, disease-driving pathways that may be targeted in rational combinations to suppress MPNSTs and/or sensitize them to immune-based therapies.

## 1. Introduction

Malignant Peripheral Nerve Sheath Tumors (MPNSTs) are an aggressive, invasive soft tissue sarcoma (STS) that affects both adolescents and adults. Tumors arise in the trunk and extremities from the transformation of Schwann cells, which are myelinating cells that surround the peripheral nerves. Like many sarcomas, MPNSTs are generally inert to chemotherapy and radiation. Despite this limitation, the only approved medical therapy for MPNST patients is a toxic, ineffective combination of doxorubicin and ifosfamide [1]. Multiple studies have shown no benefit of adjuvant chemotherapy in terms of recurrence or survival [2]. Consequently, there has been a push for targeted therapeutics to treat this disease, but none have been approved for clinical use. The only curative treatment is complete surgical resection with wide, negative margins, but that is often difficult to accomplish due to the size, amorphous shape, and location of the tumors. The 5-year survival is not good, ranging between 20 and 50% depending on the study, and, unfortunately, around half of patients receiving therapy will recur or develop metastatic disease [2,3,4]. The poor response of MPNSTs to existing treatments highlights a desperate need for new, curative therapies.

Half of all MPNSTs develop sporadically, while the other half arise in patients with neurofibromatosis Type 1 (NF1), a tumor-prone, neurological syndrome caused by germline mutations in the *NF1* gene [5]. All MPNSTs are initiated by *NF1* inactivation, although that is not sufficient for MPNST development [6]. *NF1* encodes an important tumor suppressor protein, neurofibromin, which negatively regulates oncogenic Ras by its GTPase-activating protein (GAP) activity [5]. As such, the loss of neurofibromin results in hyperactivated Ras signaling. In NF1 patients, *NF1* loss can promote the development of enlarged benign lesions called plexiform neurofibromas (PNFs). Most PNFs do not become malignant, but up to 30% will acquire other molecular alterations that promote their transformation into MPNSTs [7]. In recent years, an intermediate lesion between PNFs and MPNSTs was defined and called atypical neurofibromatous neoplasm of uncertain biological potential (ANNUBP).

**Figure 1:**
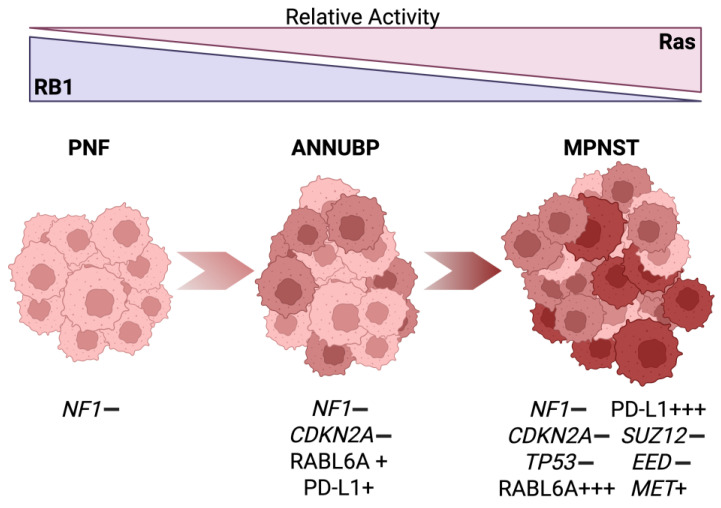
Hallmark alterations during the malignant transformation of PNFs into ANNUBPs and MPNSTs. Plexiform neurofibromas (PNFs) are initiated by the loss of *NF1*. Atypical neurofibromatous neoplasms of uncertain biological potential (ANNUBPs) are intermediate lesions characterized by an additional loss of *CDKN2A*. They also overexpress RABL6A and PD-L1 proteins. Malignant Peripheral Nerve Sheath Tumors (MPNSTs) are the fully transformed lesion, with additional losses of the *TP53*, *SUZ12*, and *EED* genes. MPNSTs also express more RABL6A, PD-L1, and MET proteins. These alterations cooperate to enhance Ras signaling and suppress RB1 activity as PNFs transform into MPNSTs. Loss, (−). Overexpression, (+); magnitude indicated by the number of (+).

**Figure 2:**
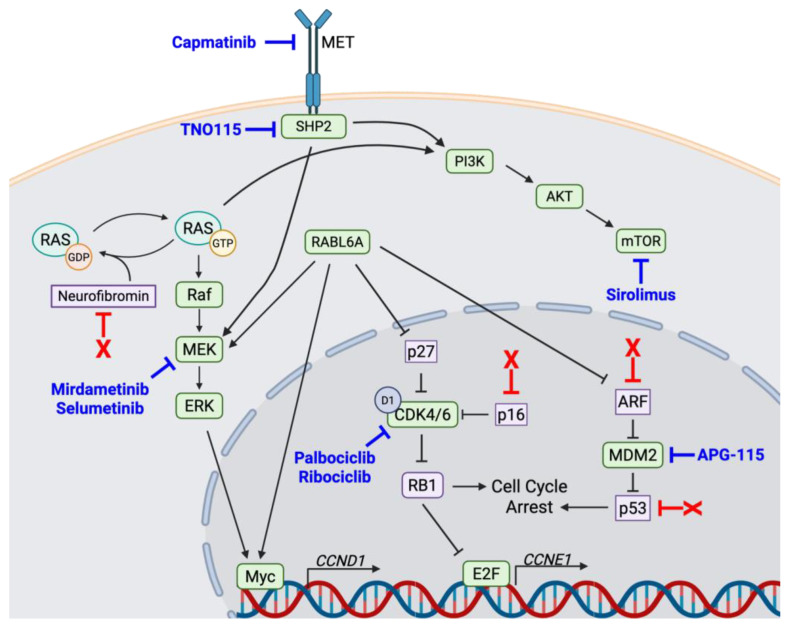
Select genetic and molecular drivers of MPNST pathogenesis. The loss of neurofibromin activates Ras, which signals through PI3K/AKT/mTOR and Raf/MEK/ERK pathways. The MET receptor (as well as other receptor tyrosine kinases like EGFR, PDGFR, and TYK2) signals through mTOR and ERK pathways through the activation of the SHP2 phosphatase. The loss of p16 activates CDK4/6, thereby inhibiting RB1. The inhibition of RB1 allows E2F to transcribe genes critical for S-phase entry, like *CCNE1*. The loss of ARF activates MDM2, destabilizing p53. p53 inactivation can also occur independently of ARF loss. RABL6A alters many pathways in MPNSTs, including the activation of MEK, Myc, CDK4/6, and MDM2 through independent mechanisms. Major tumor suppressors lost by genetic alterations in MPNSTs are designated as red X’s. Select listing of available pharmacologic inhibitors targeting MPNST drivers is shown in bolded blue text.

Several drivers of PNF-ANNUBP-MPNST transformation have been identified, although a deeper understanding of the process and the dysregulated factors involved is still needed (illustrated in Figure 1). Besides the loss of *NF1*, most ANNUBPs and MPNSTs exhibit the inactivation of *CDKN2A*, an unusual locus that encodes two powerful tumor suppressor genes, *INK4a* and *ARF* [8,9]. *INK4a* encodes the p16INK4a protein, which normally blocks cancer by inhibiting oncogenic cyclin-dependent kinases 4 and 6 (CDK4/6) and activating the Retinoblastoma 1 (RB1) tumor suppressor (Figure 2) [10,11,12,13,14]. *ARF*, which encodes the Alternative Reading Frame product of the locus, is a powerful activator of p53, although it also suppresses tumorigenesis through many p53-independent pathways [15,16,17]. The combined loss of p16INK4a and ARF in MPNSTs therefore impairs RB1 and p53-mediated tumor suppression [18,19,20,21,22].

Our laboratory discovered a new player in MPNST pathogenesis with functional ties to ARF and CDK4/6-RB1 signaling, an oncoprotein named RABL6A (Rab-like GTPase isoform 6A, also called the partner of ARF [PARF], RBEL1, or c9orf86). RABL6A binds directly to ARF and antagonizes it through undefined mechanisms [23,24]. It also upregulates the Myc transcription factor [24,25], as well as signaling through MEK-ERK [25,26] and CDK4/6 [11,27]. RABL6A is a predominantly cytosolic protein that displays a punctate pattern of localization similar to many Rab proteins that localize to endosomal membranes and control vesicular trafficking [23,28], but how that influences its oncogenic functions is not known. RABL6A is overexpressed in numerous human cancers, including common adenocarcinomas of the pancreas, lung, and breast, as well as rare malignancies like multiple types of sarcomas, and its upregulation is associated with worse patient outcomes [29,30,31,32]. Most recently, we showed that RABL6A is robustly upregulated in patient MPNSTs compared to precursor PNFs from the same individuals, and it is required for tumor survival and progression [11,12]. No pharmaceutics exist that target RABL6A, which has weak GTPase activity, but we posit that clinical drugs that inhibit RABL6A effectors, like MEK and CDK4/6, represent logical targets for developing new therapies against MPNSTs [22,33].

## 2. Dysregulated Factors in MPNSTs Provide Novel Targeted Therapeutic Options

### 2.1. MEK

While we continue to gain valuable insight into the factors driving MPNST pathogenesis, effective therapies still need to be developed. All MPNSTs are initiated through the loss of neurofibromin and the subsequent elevation of Ras activity. Like other Ras-driven cancers, MPNSTs have increased ERK phosphorylation resulting from MEK activation by Ras [34], which triggers an oncogenic transcriptional program that accelerates tumor growth (Figure 2). MEK inhibitors have had clinical success in managing PNFs. Selumetinib was the first MEK inhibitor approved for the treatment of PNFs. At 3 years, the progression-free survival of patients on selumetinib was an impressive 84% compared to 15% on the historical comparator. Excitingly, PNFs treated with selumetinib shrunk more than 20% on average during each year of treatment [35,36]. These findings led to approval for the management of PNFs with selumetinib in April of 2020. Just this year, another MEK inhibitor, named mirdametinib, was approved for treating both pediatric and adult PNFs. A phase-II trial (NCT03962543) of 58 adult and 56 pediatric cases with inoperable PNFs demonstrated good responses to MEK inhibitor therapy in more than 40% of adult patients and 52% of pediatric patients. However, there are reports of PNFs undergoing malignant transformation into MPNSTs while on MEK inhibitor therapy [37], suggesting that MEK inhibition alone is not always sufficient to suppress the benign lesions. Importantly, MEK inhibitors are not approved for the clinical management of MPNSTs.

In rare cases, MEK inhibitor monotherapy can be effective in treating MPNSTs [38], but most patients do not respond or quickly become resistant to therapy [39]. Preclinical studies with cultured MPNST cells have shown significant in vitro anti-tumor activity of MEK inhibitors in some but not all cell lines [34,40,41]. By comparison, studies of MPNST xenografts and genetically engineered mouse tumor models consistently show that in vivo MEK inhibition is ineffective. It results in a transient delay in MPNST growth, followed by the rapid development of drug resistance and tumor outgrowth [41,42,43,44]. Tumors resistant to MEK inhibitors often display elevated levels of activating phosphorylation on ERK and S6 after treatment relative to sensitive tumors [44]. Preclinical assessments of MEK inhibition combined with other targeted therapies have yielded promising results in MPNSTs. This includes synergistic anti-tumor activities of MEK therapeutics when paired with inhibitors of CDK4/6 [41]; SHP2 [42]; the mammalian target of rapamycin (mTOR) [40]; and several different receptor tyrosine kinases (RTKs), including tyrosine kinase 2 (TYK2) [45], platelet-derived growth factor receptor (PDGFR) [46], and mesenchymal epithelial transition receptor (MET) [43].

One commonality between multiple reports of MEK inhibitor resistance in MPNSTs is the adaptive upregulation of RTK activity [42,46]. Global kinase activity assays identified many RTKs activated in MPNSTs following treatment with MEK inhibitors, including MET and PDGFRβ [42]. Intriguingly, enhancing MET expression in murine Schwann cells that also lack *Nf1* drove tumorigenesis, whereas the loss of *Nf1* alone generally did not induce tumor formation [43]. Immunohistochemical (IHC) staining showed that MET-overexpressing MPNSTs had increased Ki67 positivity and ERK activation. Subsequent in vivo studies found that combined MEK and MET inhibition cooperated to combat MPNSTs in immunocompromised mice. Indeed, the combined targeting of MEK and MET abrogated ERK and MET phosphorylation better than either single agent alone in vitro and in vivo. These data reflect a larger network of interconnected signaling pathways that become dysregulated in MPNSTs in response to sustained MEK inhibition.

The upregulation of PDGFRβ following MEK inhibition offers another target to overcome MEK inhibitor resistance. After 48 h of treatment with the MEK inhibitor trametinib, five of nine MPNST cell lines tested had upregulated PDGFRβ at the protein level [46]. MPNST cell lines that were made biologically resistant to trametinib after 3 months of dose-escalated therapy also enhanced PDGFRβ expression. Ripretinib, a pan-RTK inhibitor that targets PDGRFα/β, CRAF, BRAF, and KIT, effectively sensitized MPNST cells to trametinib and cooperated to reduce cell viability, ERK phosphorylation, and ERK activity. Intriguingly, ripretinib still synergized with trametinib in *PDGFRB* KO MPNST cells, suggesting that the mechanism by which ripretinib synergizes with trametinib is not exclusive to PDGFRβ signaling.

Further studies showed that trametinib combined with ripretinib promotes the homo- and heterodimerization of BRAF and CRAF that are catalytically trapped and unable to reactivate ERK signaling [47]. Indeed, trametinib synergizes with pan- and dimer-specific-RAF inhibitors but not monomer-specific RAF inhibitors. When considered with previous studies outlining the crosstalk of MEK and MET, these data suggest that there exists a complex network of RTK-dependent and -independent mechanisms by which MPNSTs can become resistant to MEK inhibition. These findings underscore how dysregulated Ras activity can transduce signals through multiple signaling cascades, and MEK, while a biologically relevant target in MPNSTs, needs to be inhibited in concert with other hyperactivated oncoproteins to effectively combat the disease.

### 2.2. CDK4/6

After *NF1* inactivation, the second most common alteration observed in MPNSTs is the loss of *CDKN2A*, which is inactivated in about 90% of MPNSTs [48,49]. It is the only commonly altered gene shared across all types of soft tissue sarcomas whose loss is associated with worse overall survival [50]. *CDKN2A* encodes two potent tumor suppressor proteins, p16 and ARF, making it a powerful tumor-suppressing locus. p16 is an endogenous inhibitor of CDK4 and CDK6 (abbreviated as CDK4/6), two oncogenes that promote aberrant cell cycle progression through the hyperphosphorylation and inhibition of RB1. RB1 is a critical negative regulator of the E2F family of transcription factors that control the expression of CDK1, CDK2, and their cyclin partners that enable cells to progress through the S, G2, and M phases of the cell cycle [20,22]. Our group and others have shown that MPNSTs are highly sensitive to CDK4/6 inhibition, which is dependent on the expression of RB1 [51] and RABL6A [11]. RABL6A inhibits RB1 by destabilizing p27, a canonical negative regulator of CDKs 2, 4, and 6 [11,22,27]. As a result, RABL6A expression sensitizes MPNSTs to CDK4/6 inhibitors like palbociclib and ribociclib (Figure 2).

Inhibiting CDK4/6 is modestly effective at slowing tumor growth, but resistance to monotherapy develops rapidly through numerous mechanisms [52]. Combining palbociclib with inhibitors of CDK2, like dinaciclib, cooperated to suppress MPNST growth and performed better than monotherapies of each drug alone, although the toxic side effects of that combination were problematic [11]. Recent studies showed that the combination of CDK4/6 inhibitors with TNO1155, an SHP2 inhibitor (Figure 2), is highly synergistic [51]. SHP2 (Src-homology 2 domain-containing phosphatase 2) is a critical mediator of kinase pathways linking MAPK and AKT signaling to upstream receptor tyrosine kinases (RTKs) [42,53]. This suggests that the restoration of RB1 signaling and normal Ras activity levels can synergize to effectively suppress MPNST progression.

### 2.3. SHP2

Phosphatases can be either oncogenic or tumor-suppressive depending on how they control their kinase substrates in tumors. There is great interest in targeting phosphatases therapeutically, particularly those associated with receptor tyrosine kinases (RTKs) whose dysregulated signaling drives cancer through many pathways. SHP2 is one such phosphatase. Upon activation, RTKs (like EGFR, PDGFR, TYK2, and MET) recruit SHP2 to newly exposed intracellular docking sites on the cytoplasmic tail of the RTK (Figure 2). SHP2 is then phosphorylated, which induces a confirmational change to increase its phosphatase activity [53]. Once activated, SHP2 directly antagonizes NF1 by promoting Ras-GTP loading and downstream signaling through MEK [54]. This makes SHP2 an intriguing therapeutic target in Ras-driven cancers like MPNSTs. SHP2 can also promote tumor progression in an RTK-independent manner by supporting the nuclear translocation and activation of YAP/TAZ oncoproteins [53].

The Pratilas group has led most of the work studying SHP2 function and targeting in MPNSTs, particularly in combination with MEK [42] or CDK4/6 inhibitors [51]. SHP2 inhibitors alone display variable efficacy on MPNST cell viability [42]. Interestingly, SHP2 is activated, along with multiple RTKs, in MPNSTs resistant to MEK inhibitors. Subsequent studies showed that the combined inhibition of SHP2 and MEK alleviated the acquired drug resistance, synergized to inhibit ERK signaling feedback, and prevented the outgrowth of resistance tumors xenografted into nude mice. Further studies showed that SHP2 also acts through RB1. The genetic or pharmacologic inactivation of SHP2 induced RB1 activation, whereas the loss of RB1 function conferred resistance to SHP2 inhibitors [51]. These findings prompted analyses of combination therapy targeting SHP2 and CDK4/6, which caused increased cell cycle arrest and apoptosis in MPNST cells. The combination was similarly effective at suppressing the growth of patient-derived xenografts.

In summary, current evidence suggests that drugs targeting SHP2 may be most effective in treating MPNSTs when paired with inhibitors of disease-relevant kinases, like MEK or CDK4/6. However, it is noteworthy that SHP2 may also have profound effects on anti-tumor immunity in MPNSTs, as it is a negative regulator of T-cell activity [55]. In that regard, xenograft studies in immune-deficient mice have demonstrated modest efficacy of SHP2 against MPNSTs in vivo. More work in mouse models with an intact immune system is needed to understand how SHP2 inhibitors function systemically as part of anti-cancer regimens in an immune-competent setting.

### 2.4. mTOR

In addition to activating MEK, the loss of *NF1* also activates the mammalian target of rapamycin (mTOR) in a Ras- and P13K-dependent manner to support MPNST pathogenesis [56,57] (see Figure 2). mTOR can also be activated through the dysregulation of the tumor suppressor PTEN, which can be suppressed or lost in MPNSTs [58]. As a master regulator of cellular metabolism and survival, mTOR has a multitude of potential effectors. Elevated mTOR activity promotes aberrant cell cycle progression and antagonizes apoptotic signals in malignant cells and is associated with poor patient survival [59,60]. This sparked early interest in using mTOR inhibitors to combat MPNSTs. Everolimus, an mTOR inhibitor used clinically to treat other cancers, reduced the in vitro proliferation of MPNST cell lines in the nanomolar dose range [61,62]. When combined with the chemotherapeutic doxorubicin, everolimus doubled the number of apoptotic cells despite having little efficacy as a single agent. In vivo studies of everolimus activity in immunodeficient mice bearing STS26T MPNST xenografts showed that everolimus significantly delayed the time for implanted tumor cells to form detectable tumors. In established xenografts, everolimus outperformed single-agent doxorubicin, as well as erlotinib, an EGFR inhibitor, in slowing tumor progression [62].

Studies in MPNSTs and other cancers illustrate one fundamental problem with mTOR inhibitors—they are cytostatic, not cytotoxic. Moreover, the inhibition of mTOR triggers an unwanted negative feedback loop in which PI3K and AKT become activated, ultimately circumventing the anti-cancer activity of the mTOR blockade [63,64]. Acquired drug resistance is a pervasive problem for many cytostatic therapies and highlights the need for devising cytotoxic combination therapies.

## 3. Relevant Classes of Immune Therapy for MPNSTs

### 3.1. Oncolytic Viruses

**Figure 3:**
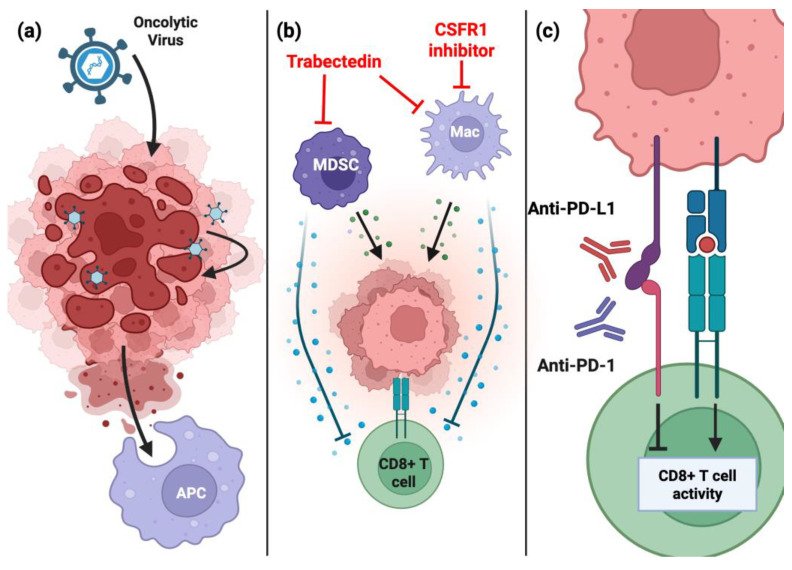
Classes of immunotherapy being tested in MPNSTs and their mechanisms of action. (**a**) Oncolytic viruses work by infecting tumor cells and lysing the tumor (depicted in red) upon replication. The spilled cytosolic contents can be taken up by antigen-presenting cells (APCs), which can then present antigens to T cells. (**b**) Myeloid cells secrete factors to promote tumor growth (green spheres) and directly inhibit T-cell cytotoxicity (blue spheres). CSFR1 inhibitors have been shown to deplete macrophages, and trabectedin has been reported to suppress both macrophages (Macs) and myeloid-derived suppressor cells (MDSCs). (**c**) Immune checkpoint blockades function by sustaining the T-cell response. PD-L1 (and other molecules) on the tumor cell (top, pink) bind receptors like PD-1 on T cells (bottom, green) to suppress CD8+ T-cell activity. Antibodies targeting either checkpoint protein can disrupt this interaction and sustain the T-cell response.

Oncolytic viruses (OVs) are naturally occurring or genetically modified lytic viruses that can replicate within and kill tumor cells in a selective manner. To use OVs for immune therapies, the challenge lies in modifying the OV such that it will not replicate efficiently in non-neoplastic cells while preserving its toxicity in the tumor cells. Initial studies of OVs found that the deletion of thymidine kinase (TK) in herpes simplex virus type I (HSV-I) allowed for tumor-specific replication. TK-deleted HSV-I prolonged mouse survival in immune-deficient, preclinical glioma models [65]. However, when studying OVs, one must acknowledge the role of host immunity. OVs kill tumor cells via two distinct mechanisms: (1) the direct lysis of tumors and (2) the activation of anti-tumor immunity through the release of cytosolic contents (Figure 3a). The local replication of OVs activates anti-tumor immunity but is subject to resistance, specifically due to the suppression of CD8+ T cells [66,67].

In MPNSTs, the efficacy of OVs is highly context dependent. A recent comparison of *Nf1-* and *Tp53*-deficient mouse MPNST allografts illustrated highly variable susceptibility to the infection, cytotoxicity, and synergy of OVs with immune checkpoint blockades, depending on the cell line. Despite the variability in infection and therapeutic success within the allografts, intratumoral injections with talimogene laherparepvec (T-VEC, a modified HSV-1 OV that is currently approved for treating unresectable melanoma) increased the infiltration of CD4+ and CD8+ T cells in all allografts. Furthermore, treatment with T-VEC was more effective when immunosuppressive factors like PD-L1, TGF-β, or macrophages were targeted in combination [68]. Other OVs, like engineered measles viruses (MV-NIS), are cytopathic to MPNST cell lines without deleterious effects on the viability of normal Schwann cells. MV-NIS is a modified measles virus that expresses a human sodium iodide symporter upon entry into the tumor cell via CD46. The insertion of the sodium iodide symporter allows for the non-invasive monitoring of infection and improves radiotherapy using iodine radioisotopes [69]. The local administration of MV-NIS induced significant tumor regression and improved cohort survival in preclinical models [70]. A phase-I clinical trial evaluating the safety of MV-NIS in recurrent or non-resectable MPNSTs was completed in September of 2024, but the results are not yet available (NCT02700230, Table 1).

### 3.2. Chimeric Antigen Receptor (CAR) T-Cell Therapy

There is rising enthusiasm about adoptive cell therapy for the treatment of MPNSTs, particularly Chimeric Antigen Receptor (CAR) T-cell therapy. CAR T-cell therapy is tailored to the patient, as the patient’s own T cells are modified to express the CAR before the reintroduction of the cells back into the individual. The CAR consists of the binding domain of an antibody specific for the antigen expressed by the tumor, which is linked to intracellular domains that exist on naturally occurring T-cell receptors, namely, the CD3zeta signaling domain and CD28 or 4-1BB as costimulatory domains [71]. In the case of cell surface proteins, CAR T cells can bind to cognate antigens directly and become activated without the need for presentation by MHC. Thus, CAR therapies may be incredibly useful in the treatment of MPNSTs and other sarcomas that reduce the expression of MHC-I proteins upon malignant transformation [72].

There are currently two ongoing trials of CAR T therapies that include MPNST patients (Table 1). Both trials are examining the safety of CAR T developed to recognize specific cell surface epitopes presented on solid tumors, such as B7-H3 and EGFR. These trials are also examining whether B cells are required for the expansion of the CAR T cells after administration by additionally providing patients with CAR T therapy directed against CD19, a cell surface marker of B cells. To limit toxicity in both trials, the modified CAR T cells express a truncated form of the target protein, which can be used to track allografted T cells or destroy them in the event of unwanted toxicities. Results are not yet available for these trials; however, CAR T-cell therapy may hold exciting promise in the treatment of MPNSTs and other sarcomas.

### 3.3. Myeloid-Focused Immunomodulatory Therapy

The immune compartment of MPNSTs is dominated by immunosuppressive myeloid cells, particularly M2-like macrophages [73]. Recently, it was reported that PLX3397 (hereafter pexidartinib) significantly reduced the number of tumor-associated macrophages (TAMs) in MPNSTs [47]. Pexidartinib is a small-molecule inhibitor of the colony-stimulating factor 1 receptor (CSFR1), which is a critical regulator of monocyte and macrophage survival through its association with interleukin (IL)-34 and macrophage colony-stimulating factor (M-CSF) [74]. In ICR/SCID xenograft mouse models, which lack T and B cells yet retain macrophages and natural killer (NK) cells, pexidartinib synergized with rapamycin, an mTOR inhibitor, to further deplete the TAM population in MPNSTs. Excitingly, TAMs were suppressed for another 3 weeks after the cessation of therapy [47]. Similar results were seen when combining rapamycin with AFS98, a CSF1R antibody blockade. Recently, a study in *Nf1-* and *Tp53*-deficient murine MPNSTs allografts implanted into immunocompetent mice showed that pexidartinib is not effective as a monotherapy; however, it synergized with the T-VEC oncolytic virus to significantly extend mouse survival [68].

Macrophages are powerful immunosuppressive cells, but they are not the only myeloid cell to take on this role (Figure 3b). In rhabdomyosarcoma (RMS), inhibiting the trafficking of myeloid-derived suppressor cells (MDSCs) alleviates resistance to and synergizes with anti-PD-1 therapy [75]. The targeting of MDSCs and macrophages with trabectedin in immune-competent MPNST allografts significantly increased mouse survival and synergized with T-VEC in a T-cell-dependent manner. Trabectedin reduced TAMs and MDSCs in MPNSTs and, when combined with T-VEC, bolstered NK and CD8+ T-cell populations. The combination also increased the number of antigen-specific CD8+ T cells, although it had no effect on the differentiation of memory T cells at the time of analysis [68]. The preclinical success of myeloid-targeting therapies has sparked enthusiasm to test them in the clinic. A phase-I clinical trial combining pexidartinib and sirolimus (an mTOR inhibitor) in unresectable sarcomas, including MPNSTs, was initiated but has been terminated due to the lack of enrollment (Table 1). While the study was not adequately powered to determine whether combining pexidartinib and sirolimus was more effective than using pexidartinib alone, the combination was quite effective in some patients. Around half of the MPNSTs were stabilized for longer than 18 weeks, although all patients eventually progressed. Perhaps a more holistic view of the myeloid compartment is required to combat MPNSTs in the clinic, as suggested by the improved preclinical efficacy of trabectedin over pexidartinib [68].

### 3.4. Immune Checkpoint Blockades

The most advanced class of immune-based treatments in the clinic for MPNSTs is called immune checkpoint blockade (ICB) therapy (Table 1). This type of therapy relies on the activation of the patient’s T cells, which are an important front-line defense of anti-tumor immunity. CD8+ T cells recognize cognate antigens presented on class I molecules and then organize an immune synapse to release cytotoxic granules that destroy the tumor cells [76]. This response is suppressed, in part, by immune checkpoint proteins, like programmed-death ligand 1 (PD-L1), to normally prevent unwanted autoimmunity by blocking the attack of healthy, self-cells. The malignant transformation of tumors often hijacks the natural function of PD-L1 and other molecules (CTLA-4, LAG3, and TIM3) to support tumorigenesis. PD-L1 is upregulated in many cancers [77], including MPNSTs [41,78,79], where its expression frequently correlates with poor clinical outcomes. Programmed cell death protein (PD-1), the receptor for PD-L1 that is expressed on T cells, suppresses T-cell activity when bound to PD-L1. Once engaged by PD-L1 or PD-L2, PD-1 activates SHP2 to reduce T-cell receptor (TCR) signaling, proliferation, cytokine production, and survival [55]. In some cancers, the development of immune checkpoint blockade (ICB) therapies has been revolutionary [80,81]. ICBs are monoclonal, therapeutic antibodies raised against specific immune checkpoint proteins. The aim of ICBs targeting PD-1 or PD-L1, for example, is to sustain the anti-tumor T-cell response by antagonizing immunosuppressive signaling (Figure 3c).

ICBs targeting PD-1 or PD-L1 have displayed great success in generating sustained anti-tumor activity for some cancer types such as renal cancer, non-small-cell lung cancer, and melanoma [82,83,84]. There are a few reports of successful anti-PD-1 therapy in MPNSTs refractory to at least two other lines of standard therapy [85,86,87,88]. However, other than a limited number of case studies, ICBs are generally ineffective in MPSNTs, as is the case with most sarcomas. SARC028, a phase-II clinical trial, studied the efficacy of pembrolizumab (anti-PD-1) in soft tissue and bone sarcomas. The efficacy of pembrolizumab depended heavily on the sarcoma subtype, ranging from a 40% response rate in undifferentiated pleiomorphic sarcoma (UPS) to a dismal 0% response in Ewings’s sarcoma [89]. The trial was expanded to enroll more UPS and dedifferentiated liposarcoma (DDLPS) patients, as they had the greatest response. Importantly, this trial did not include MPNSTs.

Currently, there are several trials examining the safety or efficacy of ICBs as a single agent or in combination with other therapies (Table 1). ANTARES is a phase-II trial of nivolumab (anti-PD-1) in multiple solid tumors, including MPNSTs. However, anti-PD-1 therapies are subject to acquired resistance when used as a monotherapy, similar to single-agent treatment modalities, albeit through different mechanisms [90]. This has prompted strategies to combine ICBs with other agents to bolster immunotherapy efficacy in MPNSTs. NCT03611868 is testing the efficacy of APG-115, an MDM2 inhibitor, plus pembrolizumab in MPNSTs and other solid tumors. Additionally, a phase-II trial is ongoing combining nivolumab with BO-112 (polyI:C dsRNA), a Toll-like receptor 3 (TLR3) agonist that has been shown to suppress MDSCs and enhance the T-cell response to the tumor in preclinical settings (NCT04420975) [91]. Finally, nivolumab is being tested in two trials in combination with another ICB, ipilimumab (anti-CTLA-4), with one trial designed for rare tumors, including MPNSTs (NCT02834013), and the other designed for newly diagnosed MPNSTs (NCT04465643). Results from these trials are not yet available, but they should be informative in guiding a path toward effectively treating MPNSTs.

## 4. The Sarcoma Microenvironment in Driving Resistance to ICB Therapies

MPNSTs harbor an immune-suppressive tumor microenvironment (TME). Like many other sarcomas [92], MPNSTs have decreased antigen presentation through the major histocompatibility complex class I (MHC-I) molecules compared to Schwann cells in the normal nerve [72]. MHC-I is vital for presenting intracellularly processed antigens to anti-tumor CD8+ T cells and natural killer (NK) cells, which become activated upon recognizing the presented neoantigen [93,94]. Generally, the downregulation of MHC-I can provide resistance to CD8+ T-cell immunity, but cells that the lose expression of class I molecules are subject to destruction by NK cells. Importantly, the downregulation of MHC-I reduces CD8+ T-cell activation, thereby reducing the efficacy of and conferring resistance to ICB therapies [92,95,96,97]. Beta-2-microglobulin (B2M), a common peptide chain in all MHC-I complexes, is transcriptionally suppressed as Schwann cells transition into PNFs [72]. As PNFs transform into MPNSTs, the expression of B2M and other proteins in the MHC-I complex is paradoxically elevated yet remains lower than that of normal Schwann cells. Furthermore, MPNSTs with higher levels of B2M expression also display more robust CD8+ T-cell infiltration.

Schwann cells, and, consequently, MPNSTs, are unique, as they also express MHC-II antigen presentation molecules, which were originally thought to be expressed only on professional antigen-presenting cells (dendritic cells, B cells, and macrophages). Similar to MHC-I, the expression of MHC-II molecules is present in MPNSTs, albeit lower than in normal tissue [98]. Although the mechanism by which MPNSTs downregulate the expression of these proteins is unknown, there is a strong correlation between the loss of Polycomb Repressor Complex 2 (PRC2) and the reduction in MHC-I and MHC-II [99]. Without sufficient antigen presentation, MPNSTs can evade recognition by T cells, functionally negating the efficacy of ICB therapies.

In addition to evading host immunity through the loss of antigen presentation molecules, MPNSTs also overexpress immunosuppressive checkpoint ligands. Of particular interest to our group is PD-L1, which we and others showed is elevated in MPNSTs compared to in normal nerve and PNFs [41,78,79]. In other cancers, oncogenic Ras promotes immunosuppression by stabilizing *CD274* mRNA, which encodes PD-L1 [100]. In melanoma, the loss of *NF1* increases PD-L1 protein expression, thereby promoting resistance to T-cell cytotoxicity [101]. While these phenomena have not been directly observed in MPNSTs, it is likely that oncogenic Ras supports the immunosuppressive MPSNT microenvironment as it does in other cancers [102]. This has sparked interest in using ICBs targeting PD-1 and PD-L1 in MPNST patients, as described earlier, but minimal success has been observed thus far, presumably reflecting the immunologically “cold” nature of MPNSTs.

Another feature contributing to the immunosuppressive MPNST microenvironment is the composition of tumor-infiltrating immune cells. The molecular profiling of MPNSTs has demonstrated that the immune compartment is largely dominated by TAMs and CD8+ T cells [103,104]. One study found that MPNSTs with higher levels of CD163+ TAMs tend to have poor survival [105], underscoring a meaningful role for macrophages in affecting MPNST progression. This is consistent with other subtypes of STS, where the abundance of immunosuppressive TAMs has been linked to ICB therapy resistance [104]. SARC028, a phase-II clinical trial of anti-PD-1 therapy in soft tissue sarcomas, found that patients responded better to ICBs if they harbored high levels of PD-1+CD8+ T cells and PD-L1+ TAMs [106]. Most of the prior research regarding ICB therapy efficacy in sarcomas was centered on T-cell biology. Currently, there is interest in understanding how other immune cells, namely, myeloid and B cells, promote or combat MPNST pathogenesis.

Sarcomas are a highly heterogenous class of cancers, each with its own unique fingerprint of tumor-infiltrating immune cells. In undifferentiated pleiomorphic sarcoma (UPS), for instance, T cells diffusely located throughout the tumor. Conversely, in rhabdomyosarcoma (RMS), T cells are located in tertiary lymphoid structures (TLSs) that are rich in B cells, plasma cells (differentiated B cells that produce antibodies), and perivascular beds. Over the past several years, there has been rising interest in the prognostic power of intratumoral B cells and plasma cells in cancer because higher fractions of these cells predict enhanced overall survival for the vast majority of human malignancies [107]. This includes several types of sarcomas [107,108,109], although MPNSTs have yet to be evaluated in this regard. Unexpectedly, the levels of intratumoral B and plasma cells are even better prognostic factors for improved patient survival than those of tumor-infiltrating T cells [107,110].

Accumulating evidence also demonstrates that elevated intratumoral B and plasma cells predict more favorable responses to ICB therapies [108,111,112,113]. This suggests that knowing the status of intratumoral B and plasma cells could guide the selection of cancer patients for treatment with immune-based therapies. Whether it is the B cells, plasma cells, or both that contribute to the improved patient survival and responses to ICB therapy remains unclear. One study in melanoma patients showed that the depletion of all B-lineage cells by anti-CD20 immunotherapy reduced tumor-associated inflammation and CD8^+^ T-cell numbers, while a higher frequency of plasmablasts in pretherapy melanomas predicted a better response to and survival following ICB therapy [113]. Another study similarly eliminated B-lineage cells in a mouse ovarian cancer model and showed that the synergistic anti-tumor effects of abemaciclib (CDK4/6 inhibitor) and anti-PD-1 therapy were lost in the absence of B cells [114]. A key limitation of both studies is that the depletion of all B cells prevents the formation of newly differentiated plasma cells, namely, plasmablasts, which not only make antibodies (albeit at lower levels than mature plasma cells) but also internalize antigens and present them to T cells (functions lost by mature plasma cells) [115,116]. To date, no study has examined the role of plasma cells in the anti-tumor response to kinase inhibitors or ICB immunotherapy.

## 5. Remodeling the Tumor Microenvironment to Bolster ICB Immune Therapy—Lessons from CDK4/6-MEK Inhibition in Other Ras-Driven Cancers Guiding Efforts in MPNSTs

With improvements in mouse models in cancer, we now have a better understanding of the tumor-intrinsic and -extrinsic effects of targeted therapy. One of the more exciting findings from these advancements includes understanding how existing therapies alter the tumor microenvironment, including the type and number of infiltrating immune cells. Recent work by the Ruscetti group highlights how the combination of CDK4/6 and MEK inhibitors synergistically and favorably alters the tumor microenvironment of Ras-driven lung cancer and pancreatic ductal adenocarcinoma (PDAC). In preclinical models of lung cancer, CDK4/6 and MEK inhibition induced cellular senescence and an inflammatory transcriptional program called the senescence-associated secretory phenotype (SASP). SASP molecules, like tumor necrosis factor alpha (TNFα) and intercellular adhesion molecule 1 (ICAM-1), promote the infiltration of natural killer (NK) cells into the tumor, where they execute their cytotoxic functions. In their Ras mutant lung cancer model, CDK4/6 and MEK inhibition prolonged mouse survival in an NK cell- and SASP-dependent manner [117].

The same research group also examined the efficacy of CDK4/6 and MEK inhibition in Ras-driven pancreatic ductal adenocarcinoma (PDAC). As in their studies of lung cancer, CDK4/6 and MEK inhibitors suppressed PDAC proliferation and induced SASP in vivo. However, in the PDAC microenvironment, an increase in CD8+ T cells, not NK cells, was induced by therapy [118]. This reflected the tissue-specific SASP molecules produced and the resulting angiogenic effects. Senescent cells displayed the activation of the transcription factor nuclear factor-kappa B (NF-κB), which stimulated an increased expression of vascular endothelial growth factor (VEGF) and the consequent reorganization of tumor vasculature, ultimately increasing the permeability of the tumors and enhancing CD8+ T-cell infiltration [118]. While tumors treated with CDK4/6 and MEK had a more abundant CD8+ T-cell population, the T cells present were largely exhausted, expressing high levels of PD-1, LAG3, and CTLA-4 [118]. Subsequent experiments combining CDK4/6 and MEK inhibitors with adjuvant anti-PD-1 therapy showed impressive synergy. Interrupting CD8+ T-cell trafficking or depleting CD8+ T cells blunted the benefits of ICB therapy, suggesting that dual CDK4/6 and MEK inhibition drives sensitivity to immunotherapy through the specific recruitment and activation of CD8+ T cells.

Our group was the first to test the combination of CDK4/6 and MEK inhibitors in MPNSTs. In MPNST cell lines, combining CDK4/6 and MEK inhibitors synergistically induced both cell death and senescence [41]. Additional evaluation in immune-deficient xenograft models reproduced the synergistic anti-tumor efficacy of the combined inhibition of CDK4/6 and MEK, unlike the vehicle and single-agent controls. Using an immune-competent model of de novo MPNSTs, developed through the Cas9-mediated inactivation of *Nf1* and *Cdkn2a*, we found that the combination of CDK4/6 and MEK inhibitors uniquely induced transient tumor regression, a phenotype not seen in immune-deficient preclinical models. Combination-treated MPNSTs regressed for 10–15 days before developing resistance and resuming growth to terminal size. An analysis of therapy-sensitive versus -resistant de novo MPNSTs demonstrated that the dual inhibition of CDK4/6 and MEK did indeed activate CD8+ T cells, as in PDAC; however, this was accompanied by a novel increase in intratumoral plasma cells [41]. As noted above, plasma cells have been correlated with positive patient outcomes [107,108,110] and responses to ICB therapies [108,111,112,113] in other sarcomas and cancer types. Indeed, the addition of anti-PD-L1 therapy to CDK4/6 and MEK inhibitors greatly increased the survival of mice bearing de novo MPNSTs, prolonged tumor regression, and cured ~10% of treated animals. These exciting findings reveal how existing clinical therapeutics, like inhibitors of CDK4/6 and MEK, can be repurposed to sensitize tumors to ICB immunotherapy and provide durable tumor control.

## 6. Conclusions

MPNSTs are one of many sarcoma subtypes for which targeted therapeutics have yet to be approved and immunotherapy trials are just beginning. The immunosuppressive tumor microenvironment of MPNSTs is an inherent barrier to successful therapy. Therefore, it is important to better understand how disease-driving alterations suppress anti-tumor immunity and how drugs targeting those pathways may reverse those phenotypes. The field is making promising strides in this direction. Dysregulated MEK and CDK4/6 signaling, among others, represent outstanding targets for combination therapies, particularly given that inhibitors of those factors can favorably remodel the MPNST immune environment to enhance sensitivity to ICB therapies. It is exciting that trials evaluating multiple ICB agents are now underway in MPNST patients, and it is expected that more trials pairing those agents with targeted therapeutics will be initiated. Many biomarkers of immunotherapy success center on T cells, but new players within the tumor microenvironment like B and plasma cells may be critical to the success of ICB and other immune-based therapies. In that regard, while the majority of work has thus far focused on ICB therapies, there are a number of different immunotherapeutic strategies being examined for utility in MPNSTs, such as oncolytic viruses, CAR T cells, and the modulation of the myeloid compartment. Further investigation into how targeted therapies control tumor growth, with a particular focus on how they modulate the immune compartment, will guide the rational development of unique drug combinations that prime MPNSTs for durable responsiveness to ICB therapies.

## Figures and Tables

**Table 1:** Clinical trials involving immune-targeting agents in MPNSTs.

NCT Number	Trial Title	Agent(s)	Phase
Oncolytic viruses			
NCT02700230	Vaccine Therapy in Treating Patients with Malignant Peripheral Nerve Sheath Tumor That is Recurrent or Cannot Be Removed by Surgery	Edmonston (Ed) strain measles virus modified to express the human thyroidal sodium iodide symporter (MV-NIS)	I
Chimeric Antigen Receptor (CAR) T-cell therapy			
NCT04483778	B7H3 CAR-T Cell Immunotherapy for Recurrent/Refractory Solid Tumors in Children and Young Adults	Anti-B7-H3 CAR-T +/− anti-CD19 CAR-T	I
NCT03618381	EGFR806 CAR-T Cell Immunotherapy in Recurrent/Refractory Solid Tumors in Children and Young Adults	Anti-EGFR CAR-T +/− anti-CD19 CAR-T	I
Myeloid-focused immunomodulatory therapies			
NCT02584647	PLX3397 Plus Sirolimus in Unresectable Sarcoma and Malignant Peripheral Nerve Sheath Tumors (PLX3397)	PLX3397 (CSFR1 inhibitor) plus sirolimus (mTOR inhibitor)	I
Combination of immune checkpoint blockades with other molecules			
NCT06638931	Agnostic Therapy in Rare Solid Tumors (ANTARES)	Nivolumab (anti-PD-1 mAb)	II
NCT03611868	A Study of APG-115 in Combination with Pembrolizumab in Patients With Metastatic Melanomas or Advanced Solid Tumors	APG-115 (MDM2 inhibitor) + pembrolizumab (anti-PD-1 mAb)	Ib/II
NCT04420975	Nivolumab and BO-112 Before Surgery for the Treatment of Resectable Soft Tissue Sarcoma	Nivolumab (anti-PD-1 mAb) + BO-112 (polyI:C dsRNA)	I
NCT02834013	Nivolumab and Ipilimumab in Treating Patients with Rare Tumors	Nivolumab (anti-PD-1 mAb) + Ipilimumab (anti-CTLA-4 mAb)	II
NCT04465643	Neoadjuvant Nivolumab Plus Ipilimumab for Newly Diagnosed Malignant Peripheral Nerve Sheath Tumor	Nivolumab (anti-PD-1 mAb) + Ipilimumab (anti-CTLA-4 mAb)	II
